# Influence of DNP Polarizing Agents on Biochemical Processes: TEMPOL in Transient Ischemic Stroke

**DOI:** 10.1021/acschemneuro.3c00137

**Published:** 2023-08-21

**Authors:** Thanh Phong Lê, Lara Buscemi, Mario Lepore, Mor Mishkovsky, Jean-Noël Hyacinthe, Lorenz Hirt

**Affiliations:** ∇ Geneva School of Health Sciences, HES-SO University of Applied Sciences and Arts Western Switzerland, Avenue de Champel 47, 1206 Geneva, Switzerland; ‡ Laboratory of Functional and Metabolic Imaging, Institute of Physics, École Polytechnique Fédérale de Lausanne (EPFL), Station 6, 1015 Lausanne, Switzerland; § Department of Clinical Neurosciences, Lausanne University Hospital (CHUV), Rue du Bugnon 46, 1011 Lausanne, Switzerland; ∥ CIBM Center for Biomedical Imaging, École Polytechnique Fédérale de Lausanne (EPFL), Station 6, 1015 Lausanne, Switzerland; ⊥ Image Guided Intervention Laboratory, Faculty of Medicine, University of Geneva, HUG, Rue Gabrielle-Perret-Gentil 4, 1211 Geneva 14, Switzerland

**Keywords:** Hyperpolarization, dDNP, TEMPOL, stroke, MRS, lactate

## Abstract

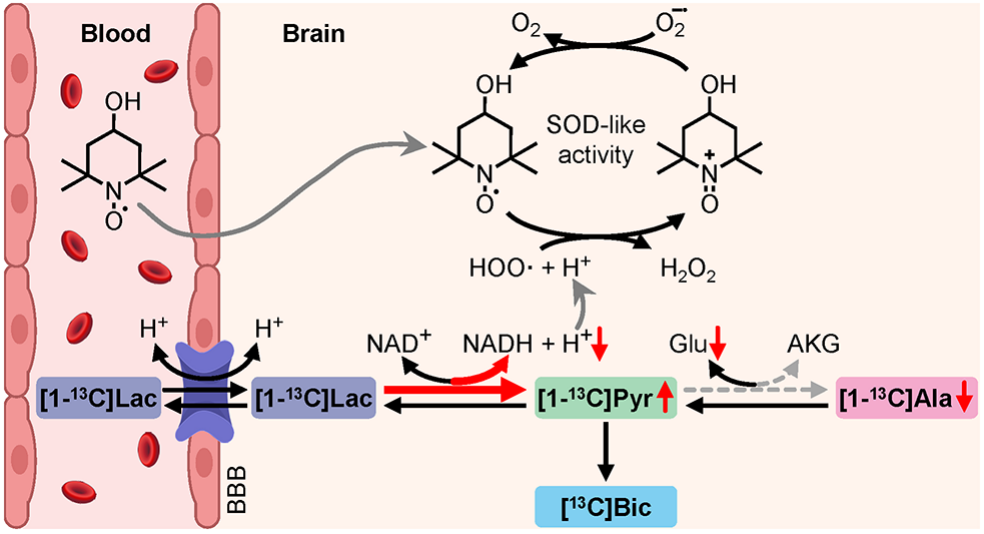

Hyperpolarization of ^13^C by dissolution dynamic nuclear polarization (dDNP) boosts the sensitivity of magnetic resonance spectroscopy (MRS), making possible the monitoring *in vivo* and in real time of the biochemical reactions of exogenously infused ^13^C-labeled metabolic tracers. The preparation of a hyperpolarized substrate requires the use of free radicals as polarizing agents. Although added at very low doses, these radicals are not biologically inert. Here, we demonstrate that the presence of the nitroxyl radical TEMPOL influences significantly the cerebral metabolic readouts of a hyperpolarized [1-^13^C] lactate bolus injection in a mouse model of ischemic stroke with reperfusion. Thus, the choice of the polarizing agent in the design of dDNP hyperpolarized MRS experiments is of great importance and should be taken into account to prevent or to consider significant effects that could act as confounding factors.

Hyperpolarization of ^13^C in labeled small molecules temporarily increases spin polarization by up to 5 orders of magnitude. Among the different hyperpolarization techniques designed to enhance nuclear magnetization,^1^ dynamic nuclear polarization (DNP) is the most versatile one. The DNP process involves the transfer of polarization from unpaired electron spins to neighboring nuclear spins through a dipolar interaction, making it possible to hyperpolarize a large variety of substrates. In a typical DNP sample, unpaired electron spins (also called polarizing agents) in the form of free radicals are homogeneously mixed with the labeled substrate of interest as frozen glassy beads. In practice, only tens of millimolar of polarizing agent are needed to hyperpolarize molars of substrate. A large variety of radicals has been proposed since the introduction of DNP,^2^ including nonpersistent UV-induced radicals^3,4^ and stable free radicals, with the latter being widely used in biological investigations.

In fact, the choice of polarizing agent has a great influence on the maximal polarization levels that can be achieved, as it influences the mechanism by which the DNP process occurs. As a rule, when directly polarizing low gyromagnetic ratio nuclei (such as ^13^C), narrow electron spin resonance (ESR) line radicals (e.g., trityl or BDPA) are preferable, and high polarization levels can be reached within several hours. On the other hand, broad ESR line radicals (e.g., nitroxyl) usually provide better performances for high gyromagnetic ratio nuclei and especially for ^1^H. Nevertheless, recent efforts for optimizing and accelerating the preparation of hyperpolarized (HP) samples while using the ubiquitous and accessible nitroxyl radicals showed that the use of microwave modulation with or without the combination of cross-polarization schemes may lead to very competitive polarization even on ^13^C-labeled compounds.^5^

The advent of the dissolution dynamic nuclear polarization (dDNP) protocol allowed for the preparation of biocompatible solutions of HP metabolic substrates. Consequently, it paved the way for novel magnetic resonance spectroscopy (MRS) applications, by enabling *in vivo* acquisitions of biochemical reactions of exogenously infused solutions of HP ^13^C-labeled tracers in real time.

Altered metabolism is a common feature of many neurological disorders, motivating extensive efforts to develop and apply HP ^13^C MRS for neuroimaging.^6−8^ Early on, proton MRS was used to report the evolution of the neurochemical profile of endogenous metabolites like lactate after ischemic brain injury.^9^ Interestingly, while it is well established that large amounts of lactate are produced in brain areas subjected to hypoxia due to reduced blood supply,^10,11^ it has been shown that exogenous lactate administration at reperfusion protects against ischemia-induced cell death and disability.^12,13^ Thus, in the context of advancing hyperpolarized MR neuroimaging and, in particular, the development of theranostic probes for ischemic stroke, we focused, as a first step, on the implementation of HP [1-^13^C] lactate as a probe for interrogating cerebral metabolism.^14,15^ We demonstrated that after transient hypoxia-ischemia injury, hyperpolarized [1-^13^C] lactate administered at a beneficial dose^12,13^ rapidly reaches the brain and gets converted into pyruvate and CO_2_.^15^

It is important to highlight that in that study the polarizing agent of choice was the widely available and affordable nitroxyl radical 4-hydroxy-2,2,6,6-tetramethylpiperidine-1-oxyl (TEMPOL). Although small amounts of TEMPOL are necessary for the DNP process and its millimolar concentration is decreased upon the dilution with the dissolution solvent, even at these low doses, it may not be biologically inactive. Indeed, TEMPOL is a radical scavenger whose antioxidant activity has been used for a long time to prevent the adverse consequences of oxidative stress and inflammation in a number of pathological conditions (see Wilcox et al. for a review^17^), which have recently incorporated SARS- and MERS-Co-Vs infections.^18^ Actually, TEMPOL has been shown to provide neuroprotection when given before or after reperfusion in animal models of brain global and focal ischemia.^19−23^ Hence, the aim of the present work was to investigate whether the presence of TEMPOL influences the cerebral metabolic readouts of an HP [1-^13^C] lactate bolus injection in a mouse model of ischemic stroke with reperfusion. In the dDNP protocol, the nitroxyl radical was replaced as a polarizing agent with the trityl radical OX063, which has not been reported for neuroprotection and produces higher ^13^C polarization^24^ in metabolic precursors. Then, the metabolic readouts of the injection of HP [1-^13^C] lactate polarized with the trityl radical were compared to that of a similar bolus spiked with TEMPOL.

The transient middle cerebral artery occlusion (MCAO) stroke procedure induces a focal ischemic lesion in the striatum, as visible in the hypersignal in representative T_2_W axial images (Figure 1A–B). Although it is only very slightly visible at 1 h post-reperfusion, the time point at which HP lactate is injected, the lesion boundaries become substantially more contrasted at 2 h post-reperfusion.

**Figure 1:**
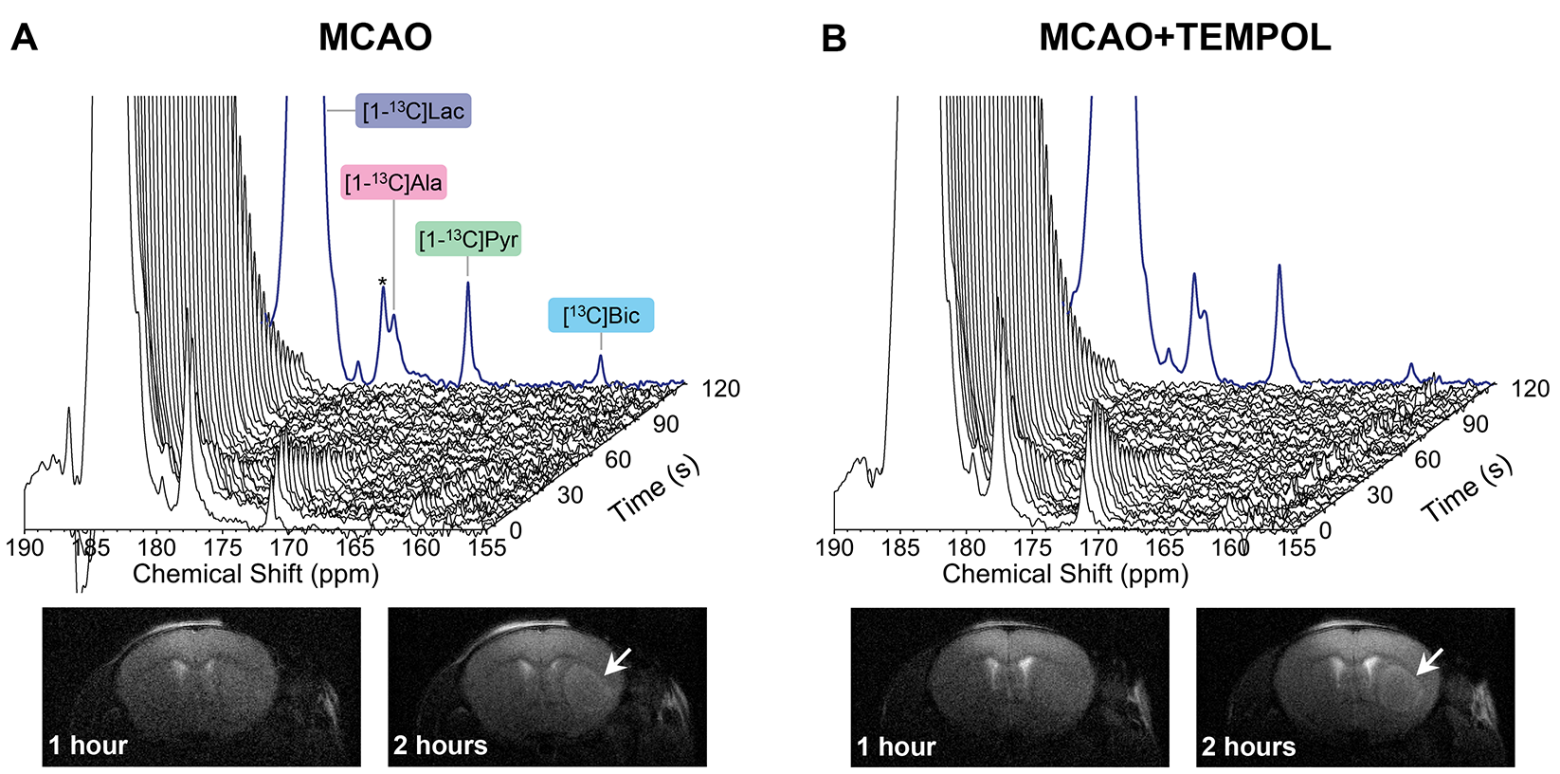
(A–B) Representative dynamic cerebral ^13^C MRS acquired after a bolus infusion of [1-^13^C] lactate (lb = 20 Hz). The summed signal from the first 120 s post-infusion is plotted in blue. The vertical scale was normalized to the height of the summed HP lactate peak. In both groups (A–B), the HP [1-^13^C] lactate (183.5 ppm) was converted into [1-^13^C] pyruvate (171.1 ppm), [1-^13^C] alanine (176.7 ppm), and [^13^C] bicarbonate (161.2 ppm). The signal observed at 177.7 ppm (*), partially overlapping with the alanine peak at 176.7 ppm, is an impurity from the stock lactate solution. Representative axial T_2_W images of the brain acquired at 1 and 2 h post-reperfusion. Images were acquired with a fast spin–echo multislice sequence (voxel size: 0.07 × 0.07 × 1 mm^3^, 4 averages). In both groups (A–B), the striatal lesion was slightly visible at 1 h post-reperfusion and clearly contrasted at 2 h post-reperfusion (white arrows).

DNP requires the use of free radicals as polarizing agents. While they are filtered out in clinical investigations, this is generally not the case in preclinical studies. Despite their low concentration in the sample after dissolution, they could still potentially interfere with the biochemical processes that are being probed. Here we show that the *in vivo* administration of the nitroxyl radical TEMPOL, even at the low dose used as polarizing agent, significantly altered the cerebral metabolic response to an HP [1-^13^C] lactate bolus injection following transient hypoxia-ischemia. As expected, immediately after the infusion of HP [1-^13^C] lactate at 1 h post-reperfusion, [1-^13^C] pyruvate, [1-^13^C] alanine, and [^13^C] bicarbonate were detected in the brain compartment (Figure 1 and Figure 2). However, the metabolite ratios, computed from the sum of the MRS signals acquired in the first 120 s post-infusion, reported distinct outputs between mice receiving HP [1-^13^C] lactate polarized with trityl radical after MCAO (to be referred to as the MCAO group) and mice receiving the bolus of HP [1-^13^C] lactate with the addition of TEMPOL after MCAO (to be referred to as the MCAO+TEMPOL group, Figure 3). While the pyruvate-to-lactate ratio (PLR, Figure 3A) of the MCAO group ((7.0 ± 1.4) × 10^–3^) was lower than that of the group which received the co-injection of TEMPOL ((10.7 ± 2.1) × 10^–3^), the alanine-to-lactate ratio (ALR, Figure 3B) was lower in the MCAO+TEMPOL group ((5.3 ± 1.0) × 10^–3^) compared to the MCAO group ((8.2 ± 1.7) × 10^–3^). The alanine-to-pyruvate ratio (APR, Figure 3D) was significantly lower in the MCAO+TEMPOL group (0.51 ± 0.11) than in the MCAO group (1.19 ± 0.20). Finally, no changes were observed in the lactate-to-bicarbonate conversion (BLR, Figure 3C). By polarizing ^13^C using OX063 we were able to double the initial polarization compared to our previous results with TEMPOL.^15^ The increased sensitivity enabled an accurate quantification of the ^13^C-bicarbonate signals.

**Figure 2:**
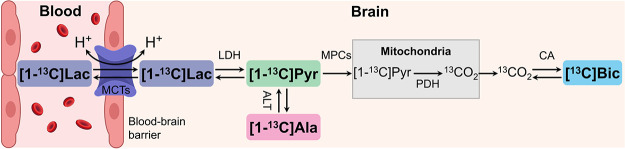
Simplified schematic of the cerebral [1-^13^C] lactate metabolism. [1-^13^C] lactate can cross the blood–brain barrier (BBB) via monocarboxylate transporters (MCTs). The intracellular [1-^13^C] lactate and [1-^13^C] pyruvate pools are exchanged via lactate dehydrogenase (LDH). [1-^13^C] pyruvate is either converted into [1-^13^C] alanine by alanine aminotransferase (ALT) or transported into the mitochondria via mitochondrial pyruvate carriers (MPCs) and then oxidized by pyruvate dehydrogenase (PDH), producing ^13^CO_2_ remaining in equilibrium with [^13^C] bicarbonate via carbonic anhydrase (CA).

**Figure 3:**
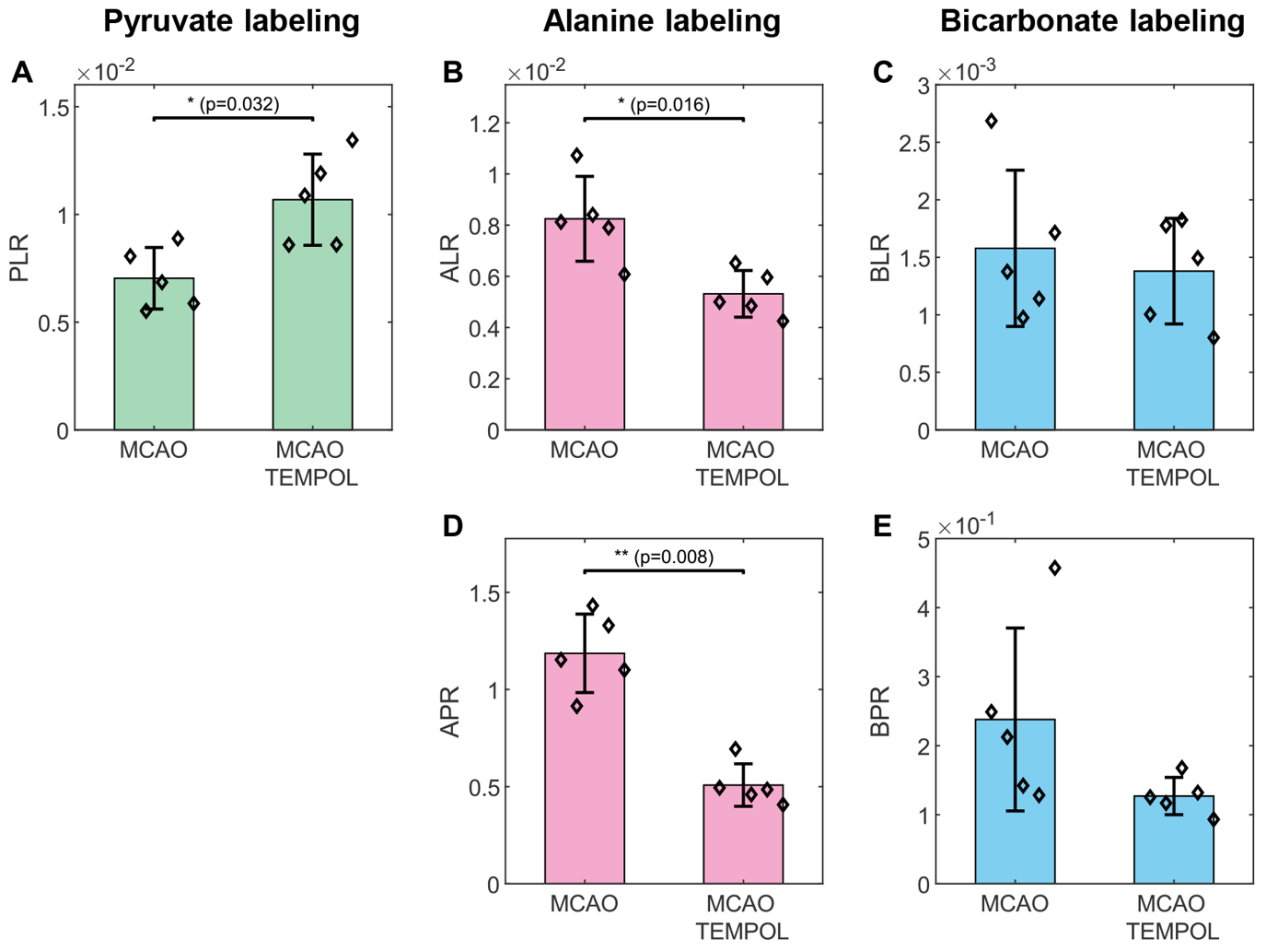
Metabolite ratios measured following injection of HP [1-^13^C] lactate. Data are displayed as the mean ± standard deviation and overlaid with individual data points (black diamonds). Pyruvate-to-lactate ratio (PLR, A), alanine-to-lactate ratio (ALR, B), bicarbonate-to-lactate ratio (BLR, C), alanine-to-pyruvate ratio (APR, D), and bicarbonate-to-pyruvate ratio (BPR, E). The PLR was significantly lower in the MCAO group compared to the MCAO+TEMPOL group (A). A lower alanine labeling (ALR) was observed in the MCAO+TEMPOL group compared to the MCAO group (B). No changes were observed in the bicarbonate-to-lactate ratio (BLR, C).

The blood flow deficit during ischemic stroke results in oxygen and glucose depletion in the affected brain region, giving rise to excitotoxicity and ionic imbalance, nitrosative and oxidative stress, as well as apoptotic cell death.^25^ The generation of toxic free radicals is even exacerbated after the restoration of blood flow, in what is known as reperfusion injury. During cerebral ischemia, superoxide anion (O_2_^·–^), which is mainly generated in mitochondria as a result of one-electron reduction of oxygen, is produced at such high levels that the ability of the natural scavenger enzyme superoxide dismutase (SOD) to dispose of it is overwhelmed. TEMPOL is a cell- and BBB-permeable compound with SOD-mimic activity that can react successively with hydroperoxyl and superoxide radicals to decompose them into H_2_O_2_ and O_2_, while consuming H^+^, acting as a self-replenishing antioxidant agent^17,26,27^ (Figure 4A). Our results highlight different PLRs between both groups of MCAO animals, being higher in those that were co-injected with TEMPOL (Figure 3A). This difference could be related to the consumption of H^+^ in the TEMPOL-mediated decomposition of reactive oxygen species, which could indirectly enhance the conversion of lactate into pyruvate by favoring a more efficient uptake of the exogenous HP [1-^13^C] lactate via the lactate/H^+^ monocarboxylate transporter (MCT) import and/or by favoring a displacement of the lactate dehydrogenase (LDH) equilibrium toward the production of [1-^13^C] pyruvate, NADH, and H^+^ (Figure 2, Figure 4B). The dose of TEMPOL used in our experiments (3.8 mg/kg) is about 5 to 50 times lower than that reported to protect at reperfusion^19,20,23^ or in other pathological conditions.^17,18,28^ Nevertheless, since TEMPOL antioxidant properties stem from a catalytic reaction, small amounts of the nitroxyl radical could be sufficient to appreciate a biological effect. In contrast, trityl radicals are typically more stable^29^ due to the electron delocalization conferred by their molecular structure. Although they react with the superoxide radical,^30^ the low dosage and noncatalytic reaction are unlikely to substantially affect the experiment outcome.

**Figure 4:**
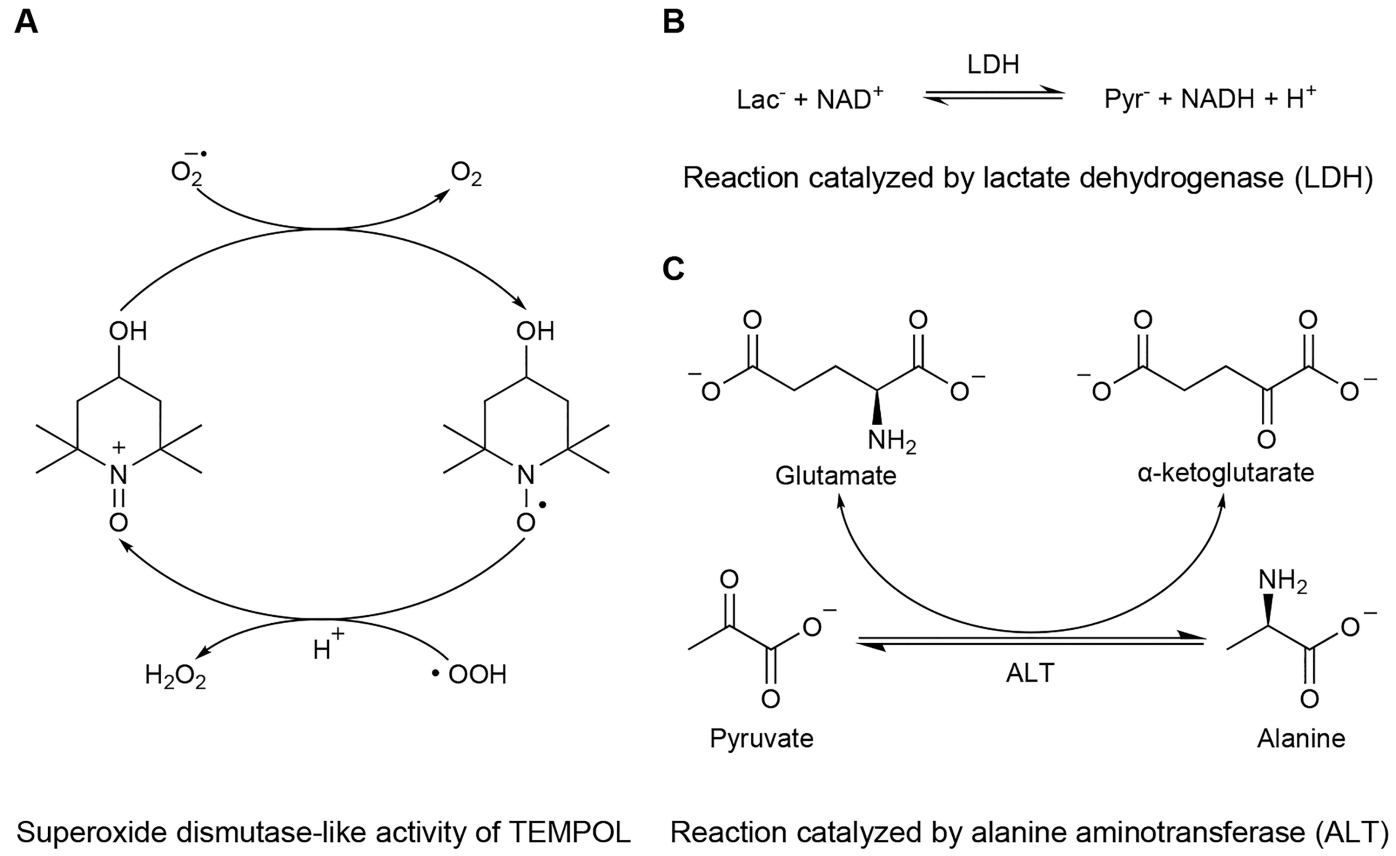
Schematic representations of the chemical reactions potentially involved in the interpretation of the measured ^13^C NMR signals, with or without TEMPOL.

Earlier experiments using HP [1-^13^C] pyruvate to study cerebral metabolism reported that [1-^13^C] alanine rather originates from peripheral tissues than from the brain.^31,32^ It is probable that the change in ALR after stroke is related to muscles around the skull, close to the coil, whose metabolism was affected by the common carotid artery ligature during surgery. However, the cerebral concentration of alanine has been reported to be steadily elevated during ischemia, an effect attenuated by TEMPOL.^22^ In the same study, TEMPOL decreased extracellular glutamate release, reduced the ischemic lesion size, and improved neurobehavioral outcomes. In the ischemic tissue, neural cells respond to oxygen and glucose deficits by rapidly depolarizing and massively releasing glutamate. This glutamate can be taken up by astrocytes, where it can be converted into alanine by alanine aminotransferase (ALT) using pyruvate as a cosubstrate.^33^ The conversion of pyruvate to alanine by ALT requiring glutamate (Figure 4C), the decrease that we observed in [1-^13^C] alanine labeling (Figure 3B and D), could thus be related to the effect of TEMPOL in decreasing the availability of glutamate to be consumed for the transamination reaction of [1-^13^C] pyruvate.

In conclusion, the administration of TEMPOL at the dose commonly used as a polarizing agent for DNP results in a significantly different cerebral metabolic response to HP [1-^13^C] lactate following transient ischemic stroke on the time scale of hyperpolarized MR examination. Our results highlight that the boost in sensitivity afforded by hyperpolarized ^13^C MRS made feasible the detection of the metabolic interference of TEMPOL. Additionally, they show that care should be taken when choosing the polarizing agent in DNP hyperpolarization experiments, as certain biologically active reagents like TEMPOL can meddle with the biochemical processes of interest. Even if used in small doses, they may have significant effects that could act as confounding factors. Not only could this be important for *in vivo* preclinical research as shown here, but it could also have some impact on new, recently developed uses of DNP HP techniques like ^13^C hyperpolarization via cross-polarization from DNP-polarized ^1^H spins, which is being applied to metabolomics studies and which so far prefers TEMPOL to achieve this cross-polarization.^34^

## Methods

### Animal Experimentation

All experiments involving mice were conducted according to federal and local ethical guidelines and were approved by the local regulatory authorities (Service de la Consommation et des Affaires Vétérinaires, Canton de Vaud, Switzerland), with license numbers VD2017.5 and VD2017.6. Male C57BL/6J mice (6 to 10 weeks, Charles River, France) were maintained in an animal facility with controlled humidity and temperature, a 12 h light/dark cycle, and free access to food and water.

### Transient Middle Cerebral Artery Occlusion (MCAO) Model of Stroke

A lesion in the left striatum was induced by transient 30 min focal cerebral ischemia as previously described.^35^ In summary, mice were kept under anesthesia with 1.5–2.0% isoflurane in 60% oxygen, and laser-Doppler flowmetry was used to monitor the regional cerebral blood flow (rCBF) through a flexible probe (Perimed AB, Sweden) glued to the skull at 1 mm posterior and 6 mm lateral from the bregma. The neck was incised, and both left common and external carotid arteries were exposed and ligated. A silicone-coated nylon monofilament (Doccol Corp., Sharon, USA) was inserted through the common carotid artery into the internal carotid artery to obstruct the left middle cerebral artery (MCA). The occluding filament was removed after 30 min to restore the blood flow. The intervention was considered successful if the rCBF remained below 20% of the baseline during occlusion and increased above 50% of the initial value within 10 min after the filament retraction. The left femoral vein was cannulated during occlusion to allow intravenous injection of the HP solution.

### Hyperpolarization

A preparation of 4.1 M sodium l-[1-^13^C] lactate (606022, Sigma-Aldrich, Buchs, Switzerland) in water/glycerol (1:1, v:v) was doped with 25 mM OX063 radical (Albeda Research, Copenhagen, Denmark). The mixture was frozen into 16 beads of about 10 μL and hyperpolarized in a 7T/1K DNP polarizer.^36^ In separate experiments, a liquid-state polarization of 33.1 ± 8.9% was measured in a 9.4T MRI scanner at the time of injection.

### Magnetic Resonance Measurements

MR measurements were performed on a 9.4T/31 cm horizontal actively shielded magnet (Magnex Scientific, Abingdon, UK) connected to a Varian INOVA spectrometer (Varian, Palo Alto, USA).

Upon reperfusion, mice were transferred into the MRI scanner with a home-built ^1^H quadrature/^13^C linear surface coil above the head, whose sensitivity profile was described in a previous study.^15^ Using the FASTESTMAP routine,^37^ static field inhomogeneity was corrected in a 3.6 mm × 6.9 mm × 4.5 mm voxel within the brain to optimize the signal quality.

Anatomical axial T_2_ weighted (T_2_W) images were acquired with a fast spin–echo sequence (effective echo time TE_eff_ = 52 ms, TR = 4000 ms, 18 mm × 9 mm FOV, 256 × 128 matrix) at the beginning of the MR scan to provide localization for the shimming voxels, as well as within 5 min of the HP injection and at 2 h post-reperfusion to assess the evolution of the striatal lesion.

At 1 h post-reperfusion, the lactate sample was dissolved in superheated D_2_O, pushed to a separator/infusion pump, and injected through the automated protocol.^36^ The injection volume was set to 450 μL, including 125 μL of dead volume, to reach a therapeutic dose of HP [1-^13^C] lactate (1.07 ± 0.14) μmol/g or (121 ± 16) mg/kg of sodium lactate. Immediately, global ^13^C HP MRS was triggered and acquired every 3 s with 30° BIR-4 adiabatic pulses. The spatial localization was provided by the coil’s sensitivity profile.^15^

### Animal Groups

Two animal groups were scanned: MCAO (*n* = 5) and MCAO+TEMPOL (*n* = 5). In the latter, a dose of 22 nmol/g or 3.8 mg/kg of 4-hydroxy-2,2,6,6-tetramethylpiperidine-1-oxyl (TEMPOL, Sigma-Aldrich, Buchs, Switzerland) was co-injected with the HP lactate bolus by adding it to the separator/infusion pump where both substances are mixed between the injection and dissolution. The TEMPOL dose was identical as when previously used as the polarizing agent.^15^

### Determination of the Injected Dose

250 μL of the remaining solution in the infusion pump was mixed with an equal volume of 80.0 mM [1-^13^C] acetate solution in D_2_O and doped with 1 mM of Gd-DO3A-butrol (Gadobutrol, Gadovist, Bayer AG, Zürich, Switzerland) to reduce the ^13^C T_1_. 1D ^13^C high resolution NMR was performed on a 400 MHz NMR spectrometer (Avance NEO, Bruker BioSpin, Fällanden, Switzerland). The integrals of the [1-^13^C] lactate and [1-^13^C] acetate peaks were compared to determine the concentration of the lactate solution.

### ^13^C MRS Data Processing

The signal from the first 120 s post-injection was summed, and then the area under the curve (AUC) of the metabolite peaks was fitted using the Bayesian Data-Analysis Software Package V4.01 (Washington University in St. Louis). The peak areas of [1-^13^C] lactate, [1-^13^C] alanine, [1-^13^C] pyruvate, and [^13^C] bicarbonate were then used to compute the metabolite ratios. Across the experimental data of this study, the concentration of the lactate solution and the weight of the animals were homogeneous.

### Statistical Analysis

Mann–Whitney U-test analyses were performed using Matlab R2021b (MathWorks, Natick, USA). A *p*-value below 0.05 was considered statistically significant. All data are presented as the mean ± standard deviation unless otherwise stated.
